# About a rare disease misdiagnosed as malignant lymphoma or tuberculosis: Kikuchi-Fujimoto’s disease

**DOI:** 10.11604/pamj.2018.31.77.16569

**Published:** 2018-10-03

**Authors:** Jawad Lahma, Zakaria Arkoubi, Reda Hejjouji, Sophia Nitassi, Ali El Ayoubi, Razika Bencheikh, Mohammed Anas Benbouzid, Abdelilah Oujilal, Leila Essakalli

**Affiliations:** 1 ENT Department, Ibn Sina University Hospital, Mohammed V University, Rabat, Morocco

**Keywords:** Kikuchi-Fujimoto disease, necrotizing histiocytic lymphadenitis, lymphadenopathy

## Abstract

Kikuchi-Fujimoto's disease KFD is a rare and benign cause of cervical lymphadenopathy. It is an anatomoclinical entity of unknown etiology. The confirmation of the diagnosis is always provided by histological lymph node study. The clinical picture sometimes evokes lymphoma or tuberculosis. The evolution is generally favorable with spontaneous healing after a few weeks. We report the case of a 26-year-old woman who had consulted for cervical lymphadenopathy associated with fever. The cervical lymph node biopsy concluded to Kikuch-Fujimoto's disease. The evolution was marked by rapid regression of lymphadenopathy under corticosteroid treatment.

## Introduction

Kikuchi-Fujimot's disease (KF) or necrotizing histiocytic lymphadenitis is a benign lymphohystiocytic disorder, first described in Japan in 1972 by Kikuchi and Fujimoto. It mainly affects young Asian women. The common reason for consultation is cervical lymphadenopathy. It is a disease of indeterminate etiology. The clinical and biological picture is not very specific and only the histopathological examination of a ganglion allows the diagnosis. We report the case of a 26-year-old woman followed for cervical lymphadenopathy due to Kikuchi's disease.

## Patient and observation

**Figure 1:**
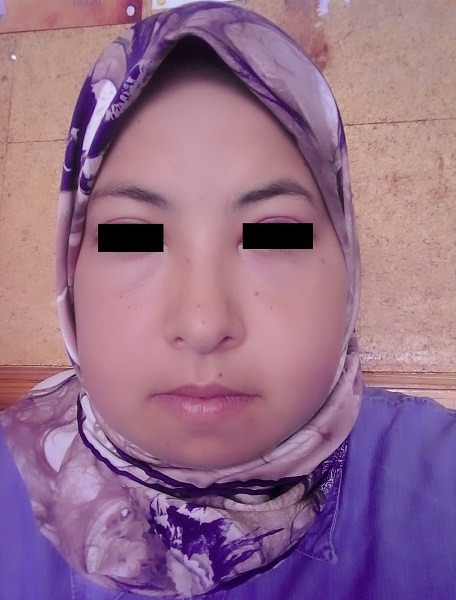
Bilateral parotid hypertrophy in our patient

**Figure 2:**
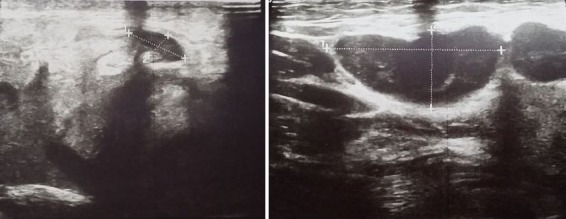
Cervical ultrasound showing cervical lymphadenopathy of hypoechogenic ovoid echostructures

**Figure 3:**
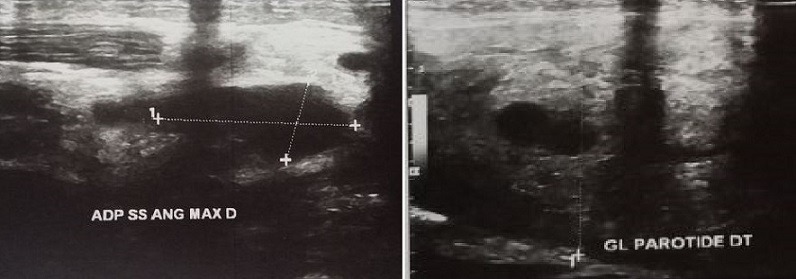
Cervical ultrasound showing inflammatory angulomaxillary adenopathy (A) and parotitis on the right (B)

A 26-year-old woman consults for parotid hypertrophy and painful bilateral cervical lymph nodes that have been evolving for a week in a febrile environment. Otherwise, she did not have a particular personal or family history or tuberculosis contagion. On the other hand, the patient was asymptomatic on the respiratory level. The clinical examination showed bilateral parotid hypertrophy (Figure 1) and several jugulo-carotid, angulo- mandibular and spinal lymph nodes, the largest of them was almost 3 cm, firm, painful and mobile. However, there was no infectious gateway and the temperature was 38.5°C. Furthermore, biological assessment showed leukopenia at 2358 with neutropenia at 1580, thrombocytopenia at 126, CRP at 4.5 and ESR at 42 mm on the first hour. Nasal endoscopy and chest x-ray were normal. Patient underwent a cervical ultrasound revealing bilateral hypertrophy of the parotid glands, predominant on the right, with poorly limbed plaques and hypoechoic nodular lesions, with intraparotid ganglia and subcutaneous soft tissue thickening in the right parotid without salivary ducts dilatation or clearly visible images of intraductal lithiasis. It is associated with bilateral jugulo-carotid, spinal, sub-mandibular and left supraclavicular adenopathy magma, wich was hypoechoic, roughly oval with largest measurement of 30 x 14 mm. This aspect concludes to bilateral parotitis prevailing on the right associated with multiple inflammatory cervical adenopathy (Figure 2, Figure 3). The patient benefited from an exploratory cervicotomy of a 1.5 cm lymphadenopathy. The histopathological examination shows the presence of numerous necrotic foci rich in clarified cytoplasmic histiocytes. Moreover, no foci of suppuration and no neutrophils or plasma cells are observed. Immunohistochemical stains with the anti-CD68 antibody and the anti-Myelo peroxidase antibody shows a positivity of this cell population. The anti-CD8 antibody remains negative. Moreover, immunohistochemical stains with anti-CD20 antibody and anti-CD5 antibody shows a bitypic distribution of the lymphoid population. The anti-CD10 antibody is strictly positive on follicle. However, the anti-Bcl2 antibody is negative. The anti-Ki67 antibody shows a positivity of more than 50% of the cell population. Thus, the morphological and immunohistochemical appearance first evoking the diagnosis of necrotizing lymphadenitis corresponding either to a Kikushi and Fujimoto disease or lupus. The patient benefited from a dosage of NAAs who returned negative coughing to Kikushi-Fujimoto disease. Therapeutic management was based on oral corticosteroid treatment with rapid clinical and radiological improvement after a few days. In addition, the patient did not present a recurrence after one year of follow up.

## Discussion

KFD is defined as necrotizing histiocytic lymphadenitis with unexplained cause [1, 2]. It has been described for the first time in Japan in 1972 by two anatomopathologists Kikuchi and Fujimoto [3, 4], it is revealed, as in our observation, by febrile lymph nodes generally cervical, firm and bulky, sometimes they are painful but never ulcerated. It is more common among Asians than Caucasians and Africans [5]. Only a few isolated North African cases are reported in the literature [6, 7]. KFD mainly affects the young adult, on average 25 to 30 years [1-7], with extremes ranging from 19 months [8] to 75 years, and a predominance of women in most series. The clinical picture is dominated by an adenopathy persistence, which is isolated in nearly half of the cases [1] and which is most often cervical (85%), uni-or bilateral. Less frequent sites have been described, superficial as in the epitrochlear, supraclavicular and inguinal level or deep as at the coelio-mesenteric or mediastinal chains [5]. They are sometimes inflammatory and painful and are accompanied by a fever in nearly a third of cases [9]. The associated clinical signs are skin lesions in 16 to 40% of cases as morbiform urticaria type. On Extranodal Manifestation we found also hepatosplenomegaly, arthralgia, night sweats, meningeal syndrome, myocardial and medullary involvement [10]. The biological disturbances are not very specific and no biological sign is pathognomonic. thus, leukopenia, sometimes lymphocytosis, rarely anemia or thrombocytopenia are observed, and liver function may be disturbed by cytolysis or cholestasis [11, 12]. Antinuclear antibodies are usually negative as for our patient [9]. Diagnostic confirmation is based on histopathological examination of adenopathy. Three histological forms are described: necrotic form (the most frequent), pseudo-tumoral proliferative form and xanthogranulomatous resorptive form [13]. The ganglionic architecture is partially respected with follicular hyperplasia, areas of necrosis in the cortical and paracortical regions characterized by cell plaques mixing histyocytes (CD 68+) including plasmocytoids cells and large CD8 T lymphoid cells in immunoblastic transformation. The absence of neutrophils and eosinophils is a constant negative sign. The two etiopathogenic hypotheses raised are infectious and autoimmune but none of them is definitively retained until today [14]. The infectious origin is suspected due to frequent association with a viral, bacterial or parasitic infection. The autoimmune hypothesis seems to be caused by the strong association of KFD with systemic lupus erythematosus. The main differential diagnosis are lymphomas, ganglionic tuberculosis, systemic lupus, toxoplasmosis, HIV infection and Kawasaki. Spontaneous evolution is usually benign with spontaneous healing without sequelae in a few weeks to a few months. Antibiotics do not influence the course of the disease [5]. Oral or bolus corticosteroids may be offered in the presence of intense clinical signs. Adenopathy surgical excision seems to accelerate healing [15]. After a few months or years of evolution, 2 to 3% of cases present recurrences, most often at the initial site, requiring histoanatomopathological confirmation [16], hence the importance of regular long-term follow-up and possibility of progression to systemic lupus erythematosus [17].

## Conclusion

KFD is a benign and rare disease in young patients that must be known and evoked by clinicians with cervical lymphadenopathy of undetermined origin, with or without prolonged fever. Histoanatomopathological examination of lymphadenopathy confirms the diagnosis. The main differential diagnoses are lymphoma and tuberculosis. The cure is constant but regular follow-up is always in order.

## Competing interests

The authors declare no competing interest.
